# Up-regulation of ceRNA TINCR by SP1 contributes to tumorigenesis in breast cancer

**DOI:** 10.1186/s12885-018-4255-3

**Published:** 2018-04-03

**Authors:** Yun Liu, Yaying Du, Xiaopeng Hu, Lu Zhao, Wenfei Xia

**Affiliations:** 1 Department of ENT, Tongji Hospital, Tongji Medical College, Huazhong University of Science and Technology, 1095 Jiefang Avenue, Wuhan, 430030 People’s Republic of China; 2 Department of General Surgery, Tongji Hospital, Tongji Medical College, Huazhong University of Science and Technology, 1095 Jiefang Avenue, Wuhan, 430030 People’s Republic of China

**Keywords:** TINCR, miR-7, KLF4, Breast cancer, Long non-coding RNA

## Abstract

**Background:**

Assembling evidences suggested that aberrant expression of tissue differentiation-inducing non-protein coding RNA (TINCR) intimately associated with variety of human cancer. However, the expression pattern and involvement of TINCR in breast cancer has not been fully investigated. Here we set out to analyze expression of TINCR in breast cancer and elucidate its mechanistic involvement in tumor incidence and progression.

**Methods:**

The expression of TINCR was determined by q-PCR. SP1 binding sites were analyzed by ChIP-qPCR. The relative transcription activity was measured with luciferase reporter assay. Cell viability was measured with CCK-8 method. Clonogenic capacity was evaluated by soft agar assay. Cell apoptosis was analyzed by Annexin V/7-AAD staining. The migration and invasion were determined by trans-well assay and wound healing. The tumor growth in vivo was evaluated in xenograft mice model. Protein expression was quantified by immunoblotting.

**Results:**

TINCR was aberrantly up-regulated by SP1, which in turn stimulated cell proliferation, anchorage-independent growth and suppressed cell apoptosis in breast cancer. TINCR silencing significantly suppressed migration and invasion in vitro and xenograft tumor growth in vivo. Mechanistically, TINCR modulated KLF4 expression via competing with miR-7, which consequently contributed to its oncogenic potential. MiR-7 inhibition severely compromised TINCR silencing-elicited tumor repressive effects.

**Conclusion:**

Our data uncovered a crucial role of TINCR-miR-7-KLF4 axis in human breast cancer.

**Electronic supplementary material:**

The online version of this article (10.1186/s12885-018-4255-3) contains supplementary material, which is available to authorized users.

## Background

Breast cancer is one of the most common malignancies in women [1]. In 2016, approximately 246,660 new cases have been diagnosed with this disease and 40,450 cancer related deaths were claimed in US [2]. Risk factors intimately associate with breast cancer incidence include female gender, obesity, never giving a birth, hormone replacement therapy for menopause et al. [3]. In addition, the environmental exposure to ionizing radiation and unhealthy life style such as lack of exercise and excessive consumption of alcohol causally link to breast cancer as well [4]. Only about 5~ 10% of all cases are due to genetic disorders such as BRCA1 and BRCA2 mutations characterized in majority of hereditary breast-ovarian cancer syndrome [5]. With advances in our knowledge about this disease at molecular level, breast cancer now is unambiguously classified into four subtypes including Luminal A (ER^+^/PR^+^/HER2^−^, grade 1 or grade 2), Luminal B (ER^+^/PR^+^/HER2^+^, grade 3), HER2 overexpression (ER^−^/PR^−^/HER2^+^) and Triple Negative Breast Cancer (TNBC, ER^−^/PR^−^/HER2^−^) [6]. The clinical management of breast cancer essentially depends on variety of disease factors including tumor stage, age and genetic causes [7]. Surgery is the most efficient option for radically curative purpose in those early-diagnosed patients, which could be more favorable while in combination with chemotherapy or radiotherapy. Hormone blocking therapy is only applicable for hormone receptor positive patients [8]. As for many other hematological malignancies and solid tumors, cell checkpoint blockade-based immunotherapy is currently under intensive investigation for clinical application in breast cancer in view of its intrinsic strong immunogenicity [9].

It’s estimated that 80% of human transcripts are not eventually translated into proteins, among which the long non-coding RNAs (lncRNA) are defined as class of RNA molecules longer than 200 nucleotides without protein-coding potential [10]. As of July 2017, there were 298 lncRNAs archived and functionally annotated in LncRNAdb (http://www.lncrnadb.org). The diverse biological functions of lncRNAs have been increasingly revealed that involved in multi-layered gene expression regulatory network [11]. In eukaryotes, lncRNAs target multiple *cis-* and *trans-* components of the transcription process, including the transcription activators, repressors, RNA polymerase II and even the DNA duplex [12]. In addition, lncRNAs also control various aspects of post-transcriptional mRNA processing in a similar way as microRNAs and snoRNAs, and potentially affect pre-mRNA splicing, transportation, translation and degradation [13]. Until recently, the crucial roles of lncRNAs in chromatin epigenetic modifications have been uncovered, which mediated imprinting, X-chromosome inactivation and telomere protections [14]. Tissue differentiation-inducing non-protein coding RNA (TINCR) is a spliced long non-coding RNA required for normal epidermal differentiation [15]. Assembling evidences suggested that aberrant expression of TINCR intimately associated with variety of human cancers [16, 17]. However, the expression pattern and involvement of TINCR in breast cancer has not been fully investigated. Here we set out to determine the expression status of TINCR and sought to elucidate its mechanistic linkage to breast cancer.

## Methods

### Patient samples

The protocol for human research was approved by the Ethics Committee of Tongji Hospital. Totally, 24 pairs of tumor (age 28–54) and adjacent normal samples were collected from the breast cancer patients enrolled in Tongji Hospital with written informed consent between March 2016 and September 2016. The diagnoses of breast cancer were histologically confirmed by three independent pathologists. The freshly collected samples were flash frozen in liquid nitrogen for RNA isolation.

### Cell culture

The immortalized human mammary epithelial cell line MCF-10A and breast cancer cell lines MDA-MB-231, MDA-MB-435, MDA-MB-453, MDA-MB-468 and MCF-7 were obtained and authenticated by the America Typical Culture Collection (ATCC). All cells were cultured in RPMI-1640 supplemented with 10% fetal bovine serum and 1% PSG (penicillin-streptavidin-glutamine). The MCF-10A was additionally supplemented with 100 ng/ml cholera toxin. The log phase cells were maintained in 37 °C humidified incubator with 5% CO_2_.

### Transfection

The MCF-7 or MDA-MB-468 cells in log phase were seeded into 6-well plate the day before transfection. The indicated plasmids were transfected using Lipofectamine 2000 in strict accordance with the manufacturer’s instructions. Transfection efficiency was evaluated by parallel assay with GFP and examined under inverted fluorescence microscope (Nikon).

### Real-time PCR

The total RNA was extracted from indicated tissue samples or cell lines using Trizol reagent (Invitrogen) following the manufacturer’s manual. The quality and quantity were determined by BioAnalyzer 2100 (Agilent) prior to further analysis. 1 μg total RNA was reversely transcribed into cDNA by PrimeScript First Strand cDNA Synthesis Kit (Clontech) according to the manufacturer’s instructions. The quantitative PCR was performed on ABI Prism 7900 HT. All primers used in this study were listed in Table 1.

**Table 1:** Primers used for qRT-PCR, qCHIP and siRNAs oligonucleotides

Primers used for qRT-PCR	
TINCR-F	TGTGGCCCAAACTCAGGGATACAT
TINCR-R	AGATGACAGTGGCTGGAGTTGTCA
GAPDH-F	GCTCTCTGCTCCTCCTGTTC
GAPDH-R	ACGACCAAATCCGTTGACTC
siRNAs/shRNA oligonucleotides	
scrambled	UUCUCCGAACGUGUCACGUdTdT
si-SP1–1	CAGCGUUUCUGCAGCUACCUUGACU
si-SP1–2	GACAGGUCAGUUGGCAGACUCUACA
siTINCR-1	UAUUCCUUCAGCCAGUACCCAGGUC
siTINCR-2	UUUCCAAGGUGGCACAGUGCUUUCC
qCHIP analysis of the *TINCR* promoter for SP1 occupancy	
BS3-F	TGACCTCGCTGATGGCTCT
BS3-R	TCAGGCGTCCGCTCCCCACT
BS1/2-F	TGAGGGGACCGTGGCA
BS1/2-R	TGGTAGCGCTTCCAGCGCGACA

### Chromatin immunoprecipitation (ChIP)

ChIP assay was performed with the SimpleChIP Assay Kits (Cell Signaling Technology). Briefly, MCF-7 cells were first treated with 37% formaldehyde for 10 min at room temperature and subjected to ultrasonication on ice. The DNA-protein complex was immune-precipitated with SP1 antibody (Cell Signaling Technologies). The bound DNA fragments were then reversely released and amplified by specific PRC reaction. The primers used in ChIP assay have been listed in Table 1.

### Dual-luciferase reporter assay

For SP1 induced overexpression of TINCR, wild-type, putative SP1 binding site deleted or mutated promoter regions were cloned into pGL4 plasmid. MCF-7 cells were transfected with luciferase reporter plasmid plus either SP1-expressing or empty vector (EV) by lipofectamine 2000. Cells were harvested 48 h later and relative luciferase activities were determined with Dual-luciferase Assay System (Promega, USA). For miR-7 regulated expression of TINCR, TINCR full length of transcript (wild-type, miR-7 target region deletion, miR-7 target region mutation) was fused to luciferase, and co-transfected MCF-7 or MDA-MB-468 cells with miR-7a/b. The luciferase activities were measured 48 h post-transfection.

### Immunoblotting

The indicated cells were lysed in RIPA buffer on ice for 30 min and cell debris were discarded via refrigerated centrifugation (12,000 rpm for 15 min). Equal amount of cell lysates was resolved by SDS-PAGE and then transferred to PVDF membrane in ice bath. The membrane was blocked with 5% milk in TBST buffer (0.05% Tween-20), and incubated with primary antibody at 4 °C overnight. After wash with TBST, the membrane was incubated with HRP-labeled secondary antibody at room temperature for 1 h. The protein bands were then visualized with enhanced chemiluminescence reagent (Cwbiotech).

### Cell proliferation assay

The relative cell viability was measured using commercial available CCK-8 Kit (Dojindo). Equal number of cells with indicated treatment was seeded into 96-well plate in triplicate for 24 h culture. Then, 10 μl of CCK-8 solution was added into each well and incubated at 37 °C for 1~ 4 h. The OD450nm was recorded by microplate reader and cell viability was calculated.

### Soft agar assay

MCF-7 and MDA-MB-468 were transfected with indicated plasmids. After 24 h culture, the cells were harvested and prepared into single-cell suspension in 2 × RPMI 1640, and then mixed with equal volume of 0.6% low-melting-point agarose (Sigma). The mixture was laid on top of solidified layer of 0.6% agarose in serum-free growth medium. The fresh and complete culture medium was added for up to 2 weeks’ culture. The visible colonies were stained with crystal violet and counted under light microscope.

### Cell apoptosis assay

The exponentially growing cells were harvested and re-suspended in HEPES buffer as single-cell solution. Staining with Annexin V-fluorescein isothiocyanate and 7-AAD (Sigma) was performed in dark at room temperature for 15 min. The apoptotic cells were determined by flow cytometry (Beckman Coulter).

### Transwell assay

The cell migration and invasion assay were performed with transwell chamber (BD). The indicated cells were starved for 24 h first, trypsinized and prepared into single-cell suspension in serum-free medium and laid on the top of polycarbonate Transwell filter (pre-coated with Matrigel (BD) for invasion assay). The lower compartment was supplied with complete culture medium containing 10% fetal bovine serum. 24 h later, the cells inside insert were completely removed with Q-tips. The migrated/invaded cells were fixed with 4% paraformaldehyde and stained with 0.025% crystal violet. For each group, the cell number was counted in five random fields under microscope to assess migratory/invasive capacity.

### Scratch healing assay

MCF-7 and MDA-MB-468 cells were first transfected with either scramble or siTINCR in 6-well plate using Lipofectamine 2000. A straight line was drawn in each well using yellow tips to stimulate a wound. The scratch width was continuously monitored and recorded at 0, 12,24 and 36 h during healing process.

### Xenograft tumor

Totally, 12 female BALB/c-nude mice (20–22 g, 4–6 weeks old) were obtained from the Shanghai Laboratory Animal Center (SLAC), and randomly divided into control and siRNA-TINCR groups after 1-week acclimatization. All experimental animals were housed in a pathogen-free environment and protocols were approved by the Committee of Animal Care and Use of Tongji Hospital. The single-cell solution was prepared and mixed with equal volume of Matrigel (BD Sciences) on ice. The mixture was cautiously inoculated subcutaneously into the right flanks of immunodeficiency mice. Tumor growth was monitored every week using and volume was determined with digital caliper according to the formula: TV (mm^3^) = length × width^2^ × 0.5. Mice were sacrificed at the endpoint indicated and subjected to macroscopic examination and weighing.

### Fluorescence in situ hybridization

TINCR subcellular localization was determined by RNA hybridization technology performed with Stellaris FISH Probes (Human TINCR with CAL Fluor-Orange 560, Human GAPDH With Quasar 670 Dye, BioSearch Technology, CA, USA) in accordance with the manufacturer’s instruction. The cells were counter-stained with DAPI and images were acquired with Zeiss LSM 800 confocal microscope.

### Statistical analysis

All data in this study were obtained from at least three independent repeats unless specified. The statistical analysis was performed with SPSS 23 software and results were presented as Mean ± standard deviation (SD). The one-way analysis of variance (ANOVA) was employed for statistical comparison. The *p* value was calculated and < 0.05 was considered as statistically different.

## Results

### SP1 stimulated TINCR overexpression in human breast cancer

**Fig. 1:**
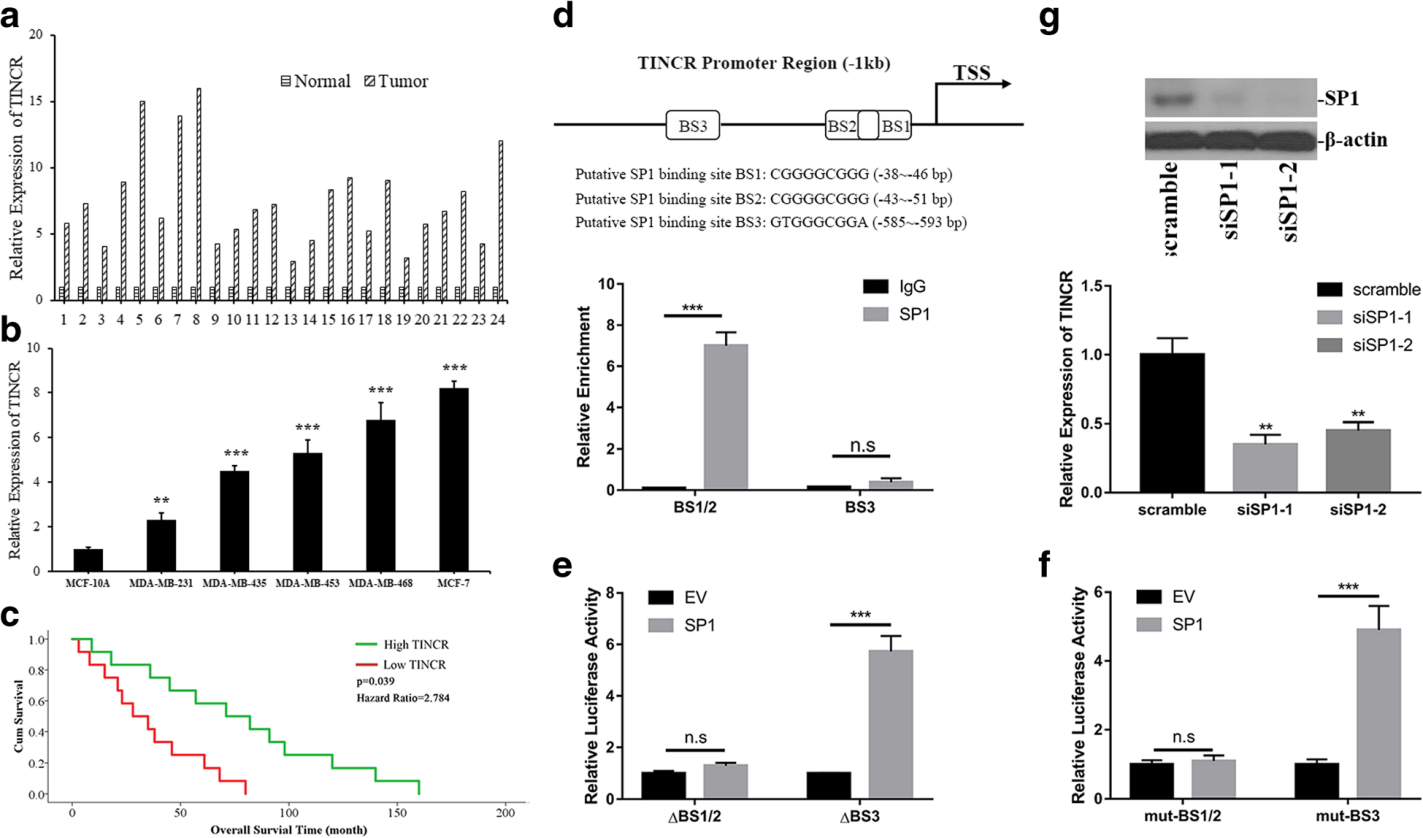
SP1 mediated overexpression of TINCR in human breast cancer. **a** The expression of TINCR was determined by real-time PCR and normalized to β-actin in human breast cancer samples (*n* = 24 pairs); **b** Relative expression of TINCR was measured by real-time PCR in human breast cancer cell line panel (*n* = 5) in comparison with immortalize human mammary epithelial cell line (MCF-10A). **p < 0.01, ***p < 0.001; **c** Kaplan-Meier curve of cumulative survival in breast cancer patients with high TINCR (*n* = 12) and low TINCR expression (n = 12). **d** The putative SP1 binding sites (BSs) across TINCR promoter predicted with PROMO (upper), the direct binding was demonstrated by SP1 enriched BS1/2 locus in ChIP assay (lower). **e** Dual-luciferase reporter assay with co-transfection of SP1 and TINCR promoter-driven luciferase plasmids carrying indicated deletion. ***p < 0.001, n.s: no significance. **f** Dual-luciferase reporter assay with co-transfection of SP1 and TINCR promoter-driven luciferase plasmids with scramble mutant in indicated regions. ***p < 0.001, n.s: no significance. **g** SP1 knockdown efficiency was evaluated by immunoblotting with β-actin as loading control. The relative expression of TINCR was determined by real-time PCR. **p < 0.01

The relative expression of TINCR was analyzed by q-PCR in 24 pairs of breast cancer tissue samples and corresponding adjacent normal tissues. As shown in Fig. 1a, TINCR was markedly over-expressed in breast cancer with 3~ 16-fold increase. We further determined the expression pattern of TINCR in panel of breast cancer cell lines. Consistent with the results from clinical samples, TINCR transcripts were significantly higher in cancer cell lines than in immortalized human mammary epithelial cell line MCF-10A (Fig. 1b). In line with previous investigations into other tumors [16, 17], our results from both clinical samples and cell lines demonstrated the consistent up-regulation of TINCR in breast cancer. Moreover, the Kaplan-Meier cumulative survival curve showed more favorable prognosis in patients with low TINCR, which supported the tumorigenic roles of TINCR (Fig. 1c).

Next, we attempted to elucidate the potential regulatory mechanism underlying aberrant overexpression of TINCR in breast cancer. Close inspection of the promoter region of TINCR identified three putative SP1 binding sites (BSs) with high G/C content (Fig. 1d, upper pane). SP1 is a zinc finger transcription factor that recognizes and binds to the GC-rich motif in promoters of multiple genes, which physiologically involved in diverse cellular processes including differentiation, growth, apoptosis, immune responses, DNA damage responses and chromatin remodeling [18]. SP1 functions as either activator or repressor of transcription via post-translational modifications such as phosphorylation, acetylation, glycosylation. Assembling evidences suggested that SP1 involved in cancer biology [19]. Here we first confirmed the direct binding of SP1 with suspected sites in TINCR promoter by ChIP assay. As shown in Fig. 1d lower pane, approximately 7-fold increase of BS1/2 fragment was enriched in SP1 immunoprecipitate in comparison with IgG. However, no obvious enrichment of BS3 was detected in our results, which clearly suggested that BS1/2 were the *cis-* elements subjected to SP1 regulation. We further consolidated this phenomenon in TINCR promoter-driven luciferase reporter assay. Either deletion or mutation introduced to BS1/2 sites significantly compromised the SP1-stimulated transcription (Fig. 1e, f), while destruction in BS3 motif showed none of effects on luciferase activity. Furthermore, siRNA-mediated knockdown of SP1 attenuated the endogenous expression of TINCR in MCF-7 cells (Fig. 1g). Taken together, our data demonstrated SP1 stimulated TINCR overexpression in breast cancer.

### TINCR knockdown inhibited malignant progression in breast cancer cells

**Fig. 2:**
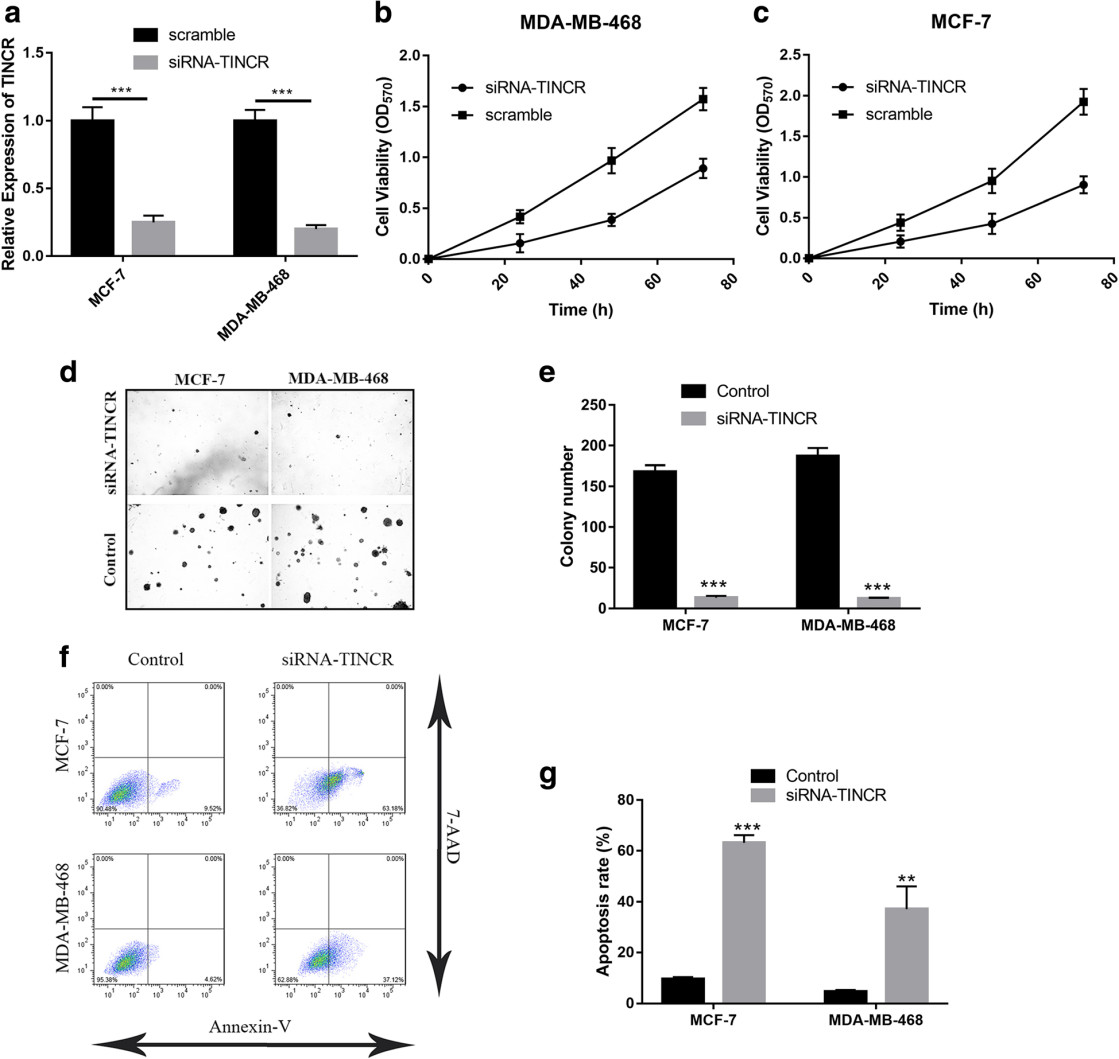
TINCR-knockdown inhibited malignant progression in breast cancer cells. **a** MCF-7 and MDA-MB-468 cells were transfected with either scramble or TINCR siRNA by lipofectamine 2000. The knockdown efficiency was confirmed by real-time PCR 48 h post-transfection. ***p < 0.001; **b**, **c** Cell viability was determined in TINCR deficient MDA-MB-468 and MCF-7 cells by CCK-8 kit. **d** Soft agar assay was performed to evaluate the anchorage-independent growth in response to TINCR silencing, the statistical results from three individual fields were shown in pane (**e**). **f**, **g** The apoptotic cells were detected with Annexin-V/7-AAD double staining method, followed by flow cytometry analysis. **p < 0.01, ***p < 0.001

Next, we sought to understand the potential oncogenic role of TINCR in breast cancer. MCF-7 and MDA-MB-468 cells were selected for mechanism study due to the relatively high expression of TINCR. SiRNA-mediated silencing of TINCR was first validated by quantitative PCR and around 70~ 80% knockdown efficiencies were achieved in both cell lines (Fig. 2a). TINCR deficiency significantly suppressed cell proliferation in MDA-MB-468 (Fig. 2b) and MCF-7 (Fig. 2c). In addition, the clonogenic capacity of MCF-7 and MDA-MB-468 was remarkably decreased in TINCR-deficient cells in comparison to the proficient counterparts (Fig. 2d, e). TINCR knockdown stimulated spontaneous apoptosis in both cell lines as well (Fig. 2f, g). In summary, our data demonstrated that inhibition of TINCR blocked malignant progression in breast cancer.

### TINCR silencing suppressed migration and invasion of breast cancer cells

**Fig. 3:**
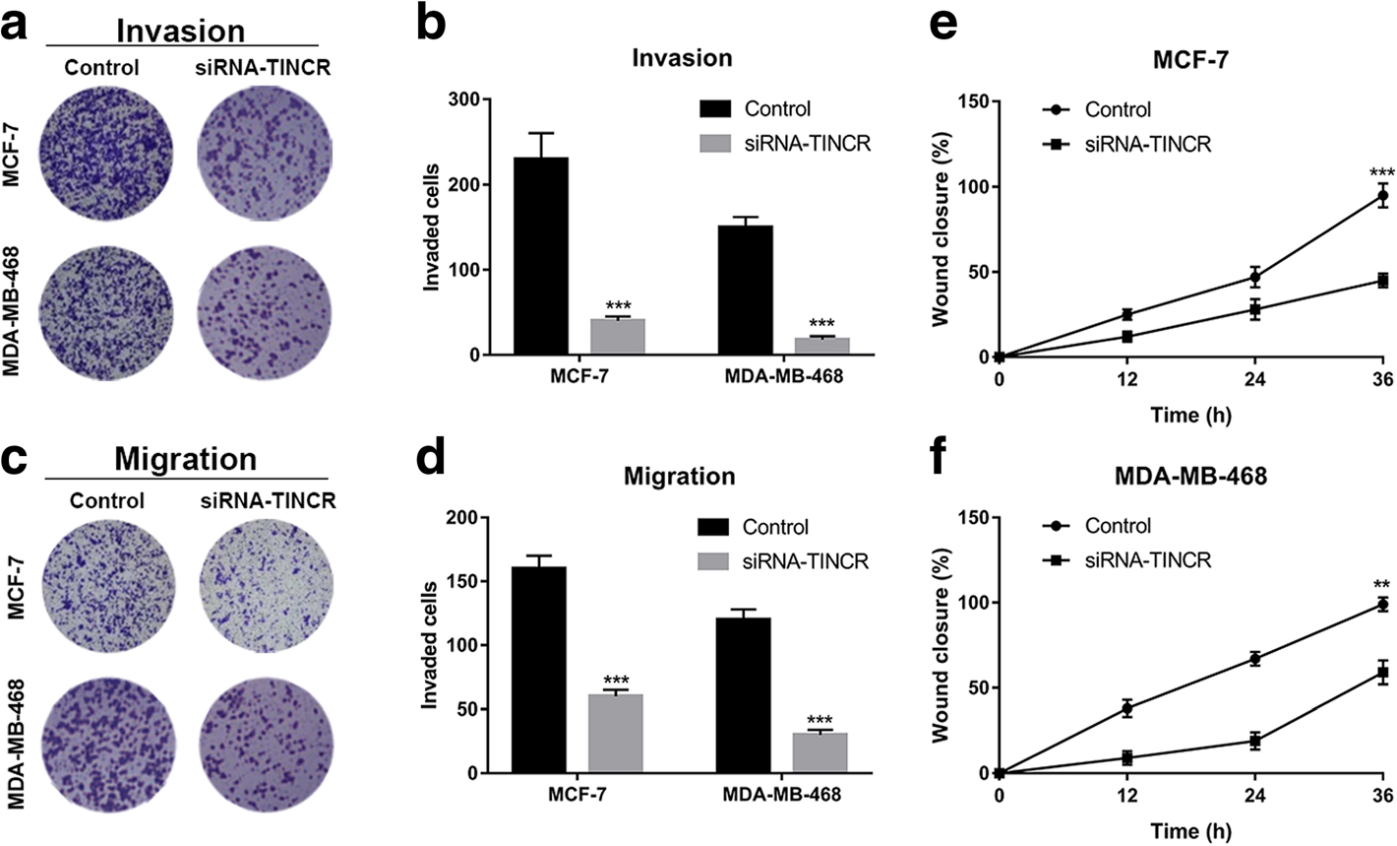
TINCR-silencing suppressed migration and invasion of breast cancer cells. **a**, **b** The invasive capacity of indicated cells were determined by Matrigel coated transwell assay. The representative images were shown in pane (**a**) and the average counting results in pane (**b**). ***p < 0.001. **c**, **d** The migratory capacities were evaluated by transwell migration assay. The representative images were shown in pane (**c**) and the average counting results in pane (**d**). ***p < 0.001. **e**, **f** Scratch-healing assay was performed to assess migration in TINCR deficient MCF-7 and MDA-MB-468 cells. The wound closure was monitored at 0, 12, 24 and 36 h respectively

Our previous data demonstrated that TINCR knockdown significantly suppressed cell proliferation, anchorage-independent growth and stimulated spontaneous apoptosis in breast cancer cells. Next, we sought to determine its impact on migration and invasion of breast cancer cells. The results acquired from transwell assay demonstrated that TINCR-silencing remarkably suppressed migratory capacity of MCF-7 and MDA-MB-468 (Fig. 3a, b). Likewise, the number of invaded cells in Matrigel-coated transwell was decreased by TINCR knockdown (Fig. 3c, d). We have performed scratch healing assay as well to validate the compromised migration elicited by TINCR silencing. Consistent with transwell assay, the wound closure was decelerated in TINCR-deficient cells in comparison with control (Fig. 3e, f), which indicated the crucial role of TINCR in maintenance of cell migration in breast cancer. Taken together, our data demonstrated that TINCR apparently involved in the metastatic process during breast tumor progression.

### TINCR silencing suppressed tumor growth in vivo

**Fig. 4:**
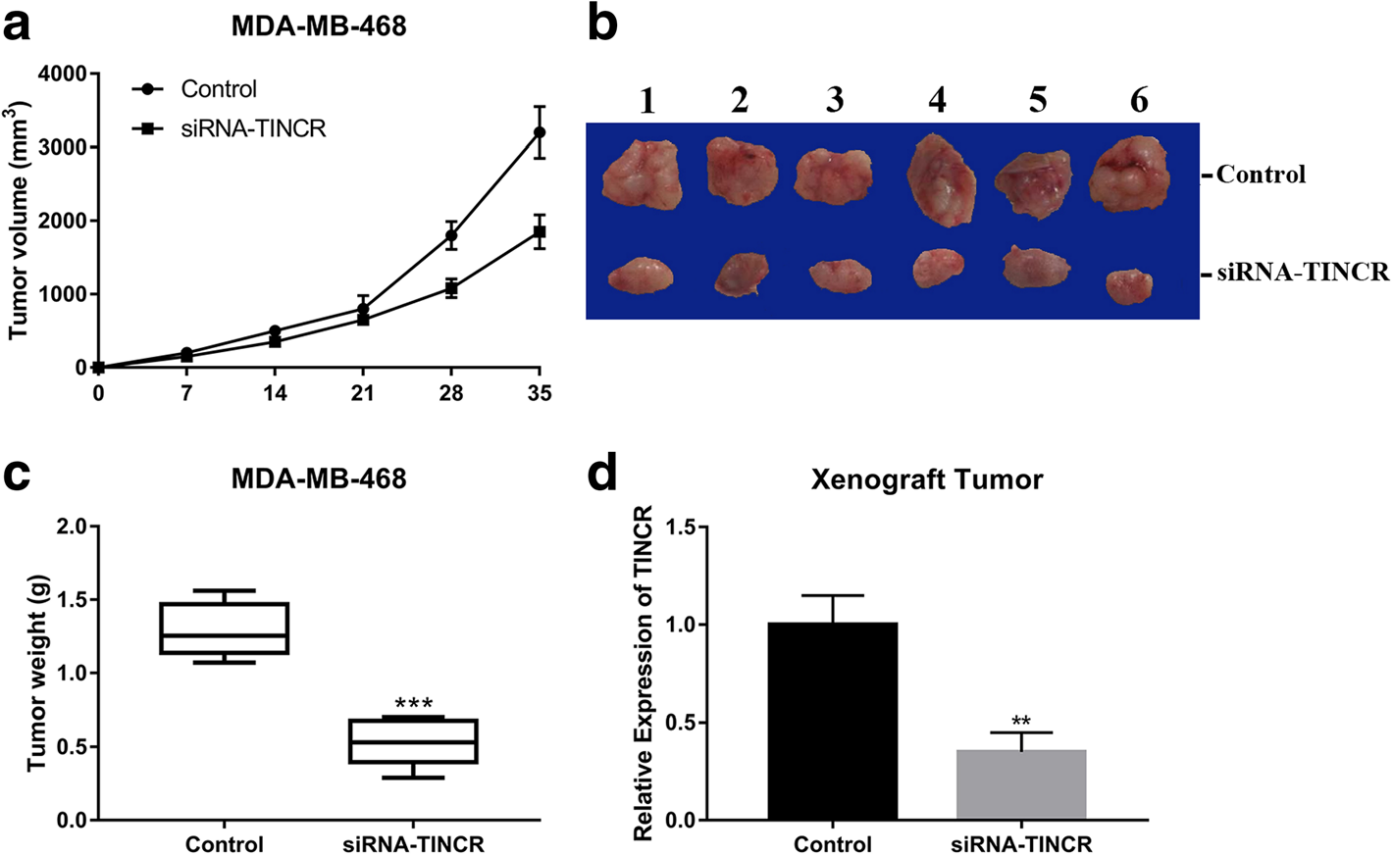
TINCR-silencing suppressed tumor growth in vivo. **a** MDA-MB-468 cells transfected with either control or siRNA-TINCR were subcutaneously inoculated into nude mice. The tumor volume was measured at 0, 7, 14, 21, 28 and 35 days post-injection respectively. **b** The macroscopic images of representative xenograft tumor 5 weeks post-inoculation. **c** The tumor weight was measured after tumor-bearing mice were sacrificed. ***p < 0.001. **d** The persistent silencing of TINCR in tumor was confirmed by real-time PCR. **p < 0.01

Noteworthily, all the above-mentioned data was acquired from cell lines in vitro. To exclude the potential artifacts associated with cell culture, we further investigated the impact on tumor growth of TINCR inhibition in xenograft tumor model. MDA-MB-468 cells transfected with either scramble or siTINCR were subcutaneously inoculated into nude mice. The continuous inspection observed significant delay in tumor growth in TINCR-knockdown mice compared to the control (Fig. 4a). The representative images of macroscopic xenograft tumor extracted from sacrificed mice revealed much smaller size in TINCR knockdown group (Fig. 4b). And the average weight of TINCR-deficient xenograft tumor was 0.5 g compared to 1.3 g in control mice (Fig. 4c). We verified the persistent knockdown of TINCR in our xenograft tumor model at the endpoint of our experiment (Fig. 4d). Therefore, we consolidated the observation that TINCR-knockdown significantly suppressed tumor growth both in vitro and in vivo.

### TINCR functioned as endogenous competing lncRNA of miR-7 and involved in KLF4 regulation

**Fig. 5:**
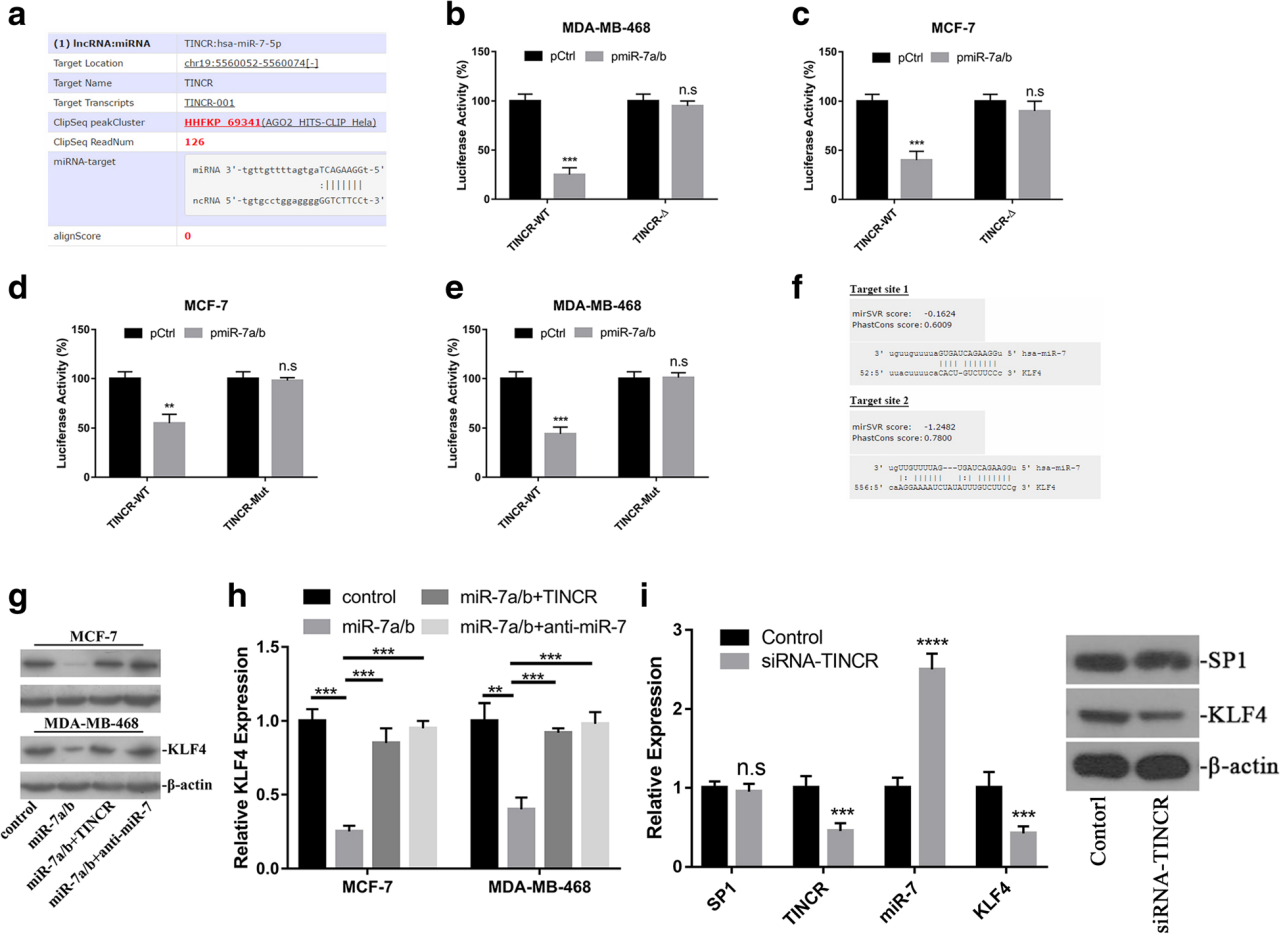
TINCR functioned as endogenous competing lncRNA against miR-7 to regulate KLF4. **a** The prediction of miR-7 seeding region in TINCR transcript using Starbase online tool. **b**, **c** Luciferase reporter assay was performed to validate the regulatory effect of miR-7 on TINCR. Either wild-type or putative binding site deleted TINCR was fused to luciferase plasmid, which was co-transfected with miR-7a/b into MDA-MB-468 and MCF-7 cells. ***p < 0.001, n.s: no significance. **d**, **e** Luciferase reporter assay was performed to validate the regulatory effect of miR-7 on TINCR. Either wild-type or putative binding site mutant TINCR was fused to luciferase plasmid, which was co-transfected with miR-7a/b into MDA-MB-468 and MCF-7 cells. **p < 0.01, ***p < 0.001, n.s: no significance. **f** The putative target sites of miR-7 in KLF4 3’UTR by microRNA online tool. **g** miR-7 negatively modulated KLF4 expression, which was antagonized by TINCR. Exogenous scramble, miR-7 a/b, TINCR or anti-miR-7 were transfected into MCF-7 (left) and MDA-MB-468 cells in combination as indicated, the relative expression of KLF4 was determined by immunoblotting. β-actin served as loading control. **h** The relative expression of KLF4 was measured by real-time PCR in indicated cells. **p < 0.01, ***p < 0.001. **i** The SP1-TINCR-miR-7-KLF4 axis was analyzed by q-PCR (left pane) and western blotting (right pane) in xenograft tumor. n.s: no significance, **p < 0.01, ***p < 0.001

The accumulative evidences suggested that long non-coding RNA might function as endogenous competing RNA against miRNAs. Here we sought to investigate the possible molecular events underlying oncogenic activity of TINCR along this direction. We employed LncRNABase online algorithm to predict the candidate miR with the potential to directly compete with TINCR, and identified miR-7 as the top one in the putative targets list. The alignment between TINCR and miR-7 was shown in Fig. 5a. Range of investigations have uncovered the important roles of miR-7 in human malignancies with increased list of target genes have been identified [20–27]. In line with previous observations, the direct binding of miR-7 with TINCR was experimentally validated in our luciferase reporter assay. The exogenous introduction of miR-7 caused about 75% reduction in MDA-MB-468 and 60% reduction in MCF-7 of TINCR-fused luciferase activity (Fig. 5b, c). We further experimentally validated the putative binding sites of miR-7 on TINCR transcript. Either deletion or mutation introduced into the suspected regions of TINCR abolished miR-7-inhibited luciferase activities (Fig. 5b, c, d, e). Consistent with its physiological role as endogenous competing RNA, the RNA hybridization results clearly demonstrated the predominant localization of TINCR in cytoplasm (Additional file 1: Figure S1). Taken together, our data suggested that TINCR might function as molecular sponge of miR-7, which eventually contributed to its oncogenic activity.

Kruppel Like Factor 4 (KLF4) is a zinc finger protein with physiological function as transcription factor. Several studies implied that KLF4 was the potential target of miR-7. Along this line, here we further investigated the potential that TINCR modulated KLF4 expression via competing with miR-7. The putative target site in 3’UTR region of KLF4 aligned with miR-7 seed region based on the micrRNA.org online prediction was showed in Fig. 5f. Exogenous introduction of miR-7a/b remarkably inhibited endogenous KLF4 in our immunoblotting results in both MCF-7 and MDA-MB-468 cells. This inhibitory effect was readily reversed by co-introduction of either TINCR or miR-7 specific inhibitor (Fig. 5g). The regulation of KLF4 by TINCR was evaluated at transcription level as well. Consistently, the transcripts of KLF4 were decreased in response to miR-7a/b, and subsequently restored by ectopic TINCR or miR-7 inhibitor treatment (Fig. 5h). The expression status of SP1-TINCR-miR-7-KLF4 was further characterized in xenograft tumor at both transcriptional and translational level (Fig. 5i), which definitely consolidated our in vitro observations.

### miR-7 inhibition abrogated TINCR-silencing elicited tumor suppressive effect

**Fig. 6:**
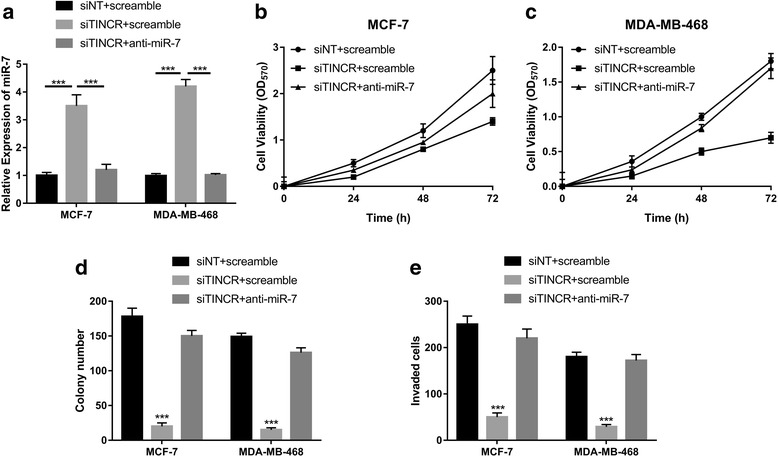
miR-7-inhibition abrogated TINCR-silencing elicited tumor suppressive effect. **a** The relative expression of miR-7 in response to TINCR knockdown or miR-7 specific inhibitor was determined by real-time PCR in both MCF-7 and MDA-MB-468 cells. ***p < 0.001. **b**, **c** The suppressive effect on cell proliferation elicited by TINCR knockdown was relieved by miR-7 inhibition. The cell viability was monitor at 0, 24, 48 and 72 h respectively using CCK-8 assay. **d** Colony formation assay was performed to determine the anchorage-independent growth of indicated cells. ***p < 0.001. **e** The invasive capacity was assessed by Matrigel coated Transwell assay. ***p < 0.001

Our previous data suggested that TINCR functioned as competing endogenous RNA (ceRNA) to compete with miR-7 in regulation of KLF4. Next, we sought to determine the extent that this regulatory axis was involved in the oncogenic activity of TINCR in breast cancer. The endogenous expression of miR-7 in response to TINCR knockdown was analyzed in both MCF-7 and MDA-MB-468 cells. As shown in Fig. 6a, TINCR-silencing induced more than 3-fold increase of miR-7, which was completely abrogated by co-treatment with miR-7 inhibitor. Consistent with previous results, TINCR-knockdown significantly suppressed breast cancer cell proliferation, while simultaneous inhibition of miR-7 in this setting almost abolished this effect (Fig. 6b, c). Similarly, the reduction in colony formation capacity elicited by TINCR silencing was readily restored while miR-7 was specifically inhibited (Fig. 6d). The inhibitory effects on invasive behavior of breast cancer cells imposed by TINCR-knockdown was almost abrogated as well (Fig. 6e). Our data unambiguously demonstrated that miR-7 predominately involved in oncogenic activity of TINCR in breast cancer.

## Discussion

TINCR is a critical modulator required for normal epidermal differentiation via regulation of array of genes involving in this physiological process [28]. More recently, the potential roles of TINCR in tumor biology have been cumulatively uncovered in variety of human malignances. For instance, Xu et al. demonstrated that E2F1 stimulated TINCR/STAU1/CDKN2B signaling axis exacerbated gastric cancer progression [16]. Zheng et al. proposed that genetic variation of TINCR contributed to the susceptibility and progression of colorectal cancer [29]. A genome-wide lncRNA microarray profiling performed by Zhang et al. has identified circulating TINCR as molecular marker for gastric cancer [30]. Xu et al. reported that SP1-induced upregulation of TINCR regulating apoptosis by affecting KLF2 mRNA stability in gastric cancer [17]. In esophageal squamous cell carcinoma, TINCR was reported to be up-regulated and promote tumorigenesis as well [31]. However, another study from Li group displayed the anti-tumor activity of TINCR in colorectal cancer, wherein loss of TINCR expression promoted proliferation, metastasis through activating EpCAM cleavage [32]. Until now, the expression and potential linkage to tumorigenesis of TINCR in breast cancer has not been fully investigated. Noteworthily, while we prepared our manuscript, Xu et al. identified the oncogenic TINCR along with a cluster of lncRNAs were upregulated in breast cancer via analysis of GEO and TCGA databases [33], which definitely consolidated our finding.

Here we for the first time demonstrated over-expression of TINCR in both breast cancer tissues and cell lines. High level of TINCR in breast cancer patients significantly associated with relatively poor prognosis. Moreover, with the aid of bioinformatics tool, here we proposed that SP1 specifically modulated TINCR expression via direct binding to its promoter region. We further experimentally validated the putative *cis-* elements for SP1 in TINCR. TINCR silencing significantly inhibited cell proliferation, anchorage-independent growth, and most notably, stimulated simultaneous apoptosis in vitro. We also evaluated the potential contribution of TINCR to breast tumor metastasis. In our transwell and scratch healing assays, TINCR-knockdown remarkably compromised the migratory and invasive capacity. The pro-tumor activity of TINCR was further demonstrated in xenograft tumor mouse model.

Assembling evidences suggested that lncRNA could function as ceRNA to sponge miRs in either physiological or pathological conditions, especially in variety of human cancers [34]. For example, lncRNA-PVT1 promoted pancreatic cancer cells proliferation and migration through acting as a molecular sponge to regulate miR-448 [35]. TP73-AS1 has been demonstrated to promote breast cancer proliferation through miR-200a-mediated TFAM inhibition [36]. Intriguingly, an integrated analysis of long non-coding RNA competing interactions in 361 gastric cancer indicated that TINCR might function as ceRNA with multiple potential miRNAs [37]. In line with this notion, in this study we performed bioinformatic analysis to identify miR-7 as one of TINCR targets. Our data further experimentally confirmed the direct interaction between miR-7 and TINCR, which underlaid its competitive regulation of miR-7 target genes.

MiR-7 is conventionally considered as a tumor suppressor miRNA in variety of human malignancies including breast cancer [24], brain cancer [21], liver cancer [38], colon cancer [26]. However, the opposite conclusion emerged as well in especially lung cancer [39], suggested both oncogene and tumor suppressor roles of miR-7 probably in an organ context-dependent manner. The candidate target genes of miR-7 involving in tumor biology have been extensively identified and systematically reviewed by Gu et al. [20]. For instance, miR-7 has been demonstrated to function as a tumor-suppressor gene in pancreatic carcinoma via regulation of ILF2 [40]. MiR-7 also suppressed cell proliferation and induced apoptosis of breast cancer cells predominately by targeting REGγ [25]. Moreover, miR-7 was shown to arrest cell cycle in G1 phase by directly targeting CCNE1 in human hepatocellular carcinoma cells [27]. Most notably, several studies indicated that miR-7 functioned as tumor suppressor gene via direct regulation of KLF4. Okuda et al. reported that miR-7 capable of suppressing brain metastasis of breast cancer stem-like cells by modulating KLF4 [23]. Chang et al. reported that miR-7 inhibited the tumorigenesis and stemness of prostate cancer via repressing KLF4/PI3K/Akt/p21 pathway [41]. On the other hand, inhibition of miR-7 was shown to promote angiogenesis in human umbilical vein endothelial cells by up-regulating VEGF via KLF4 [22]. Consistent with these observations, our data suggested that inhibitory effect on KLF4 expression of miR-7 was significantly mitigated by TINCR in breast cancer via competitive mechanism.

In summary, in this study we characterized TINCR overexpression regulated by SP1 transcription factor. High level of TINCR in turn competed with miR-7, and stabilized and promoted KLF4 expression, which consequently contributed to the oncogenic activity of TINCR. Our results clearly demonstrated the critical role of TINCR in tumorigenesis and metastasis-related malignant behavior in breast cancer, and predominant role of miR-7 in mediating this effect. Most importantly, our data suggested that either specific inhibition of TINCR or complement with miR-7 likely held great promise for breast cancer therapeutics.

## Conclusions

Our data suggested a crucial role of TINCR-miR-7-KLF4 axis in human breast cancer and up-regulation of ceRNA TINCR by SP1 contributes to tumorigenesis in breast cancer.

## Additional file

Additional file 1: **Figure S1** TINCR predominantly located in cytoplasm. Subcellular localization of TINCR was analyzed RNA hybridization with the specific Stellaris RNA FISH probes followed by confocal microscope imaging. TINCR was detected in red channel, while cytoplasmic GAPDH transcript was detected in green channel. The nuclei were counter-stained with DAPI. (JPEG 29 kb)

## Abbreviations

CeRNAs: Competing endogenous RNAs
ChIP: Chromatin immunoprecipitation
KLF4: Kruppel Like Factor 4
LncRNA: Long non-coding RNAs
ATCC: America Typical Culture Collection
PSG: Penicillin-streptavidin-glutamine
TINCR: Tissue differentiation-inducing non-protein coding RNA
