# Supplementary figures and images for: Up-regulation of ceRNA TINCR by SP1 contributes to tumorigenesis in breast cancer
Source: BMC Cancer. 2018 Apr 3;18:367. doi: 10.1186/s12885-018-4255-3 (PMC5883880; doi:10.1186/s12885-018-4255-3)

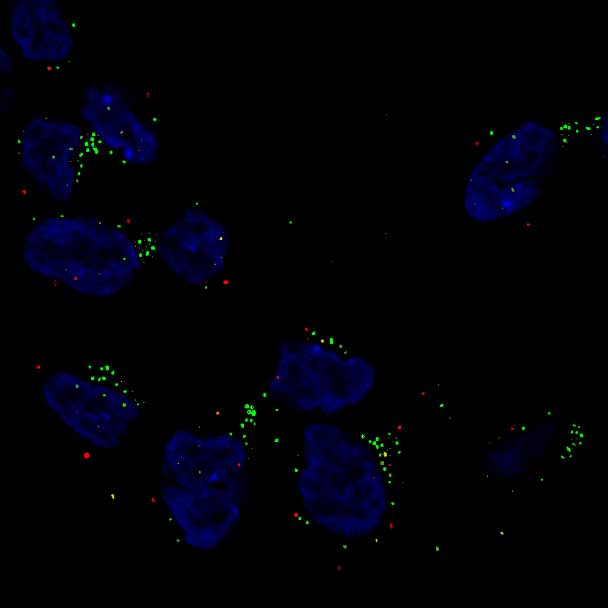

Supplement: Supplementary file 1 — Figure S1 TINCR predominantly located in cytoplasm. Subcellular localization of TINCR was analyzed RNA hybridization with the specific Stellaris RNA FISH probes followed by confocal microscope imaging. TINCR was detected in red channel, while cytoplasmic GAPDH transcript was detected in green channel. The nuclei were counter-stained with DAPI. (JPEG 29 kb) [file 12885_2018_4255_MOESM1_ESM.jpg]
